# Correlation Between Serum 25-Hydroxyvitamin D Levels in Albuminuria Progression of Diabetic Kidney Disease and Underlying Mechanisms By Bioinformatics Analysis

**DOI:** 10.3389/fendo.2022.880930

**Published:** 2022-05-12

**Authors:** Bin Huang, Wenjie Wen, Shandong Ye

**Affiliations:** ^1^ Department of Endocrinology, The First Affiliated Hospital of University of Science and Technology of China (USTC), Division of Life Science and Medicine, University of Science and Technology of China, Hefei, China; ^2^ Department of Life Sciences and Medicine, University of Science and Technology of China, Hefei, China

**Keywords:** 25-hydroxyvitamin D, diabetic kidney disease (DKD), albuminuria, progression, M2 macrophage infiltration

## Abstract

**Aim:**

This study aimed to assess the correlation between serum concentration of 25-hydroxyvitamin D and albuminuria progression of diabetic kidney disease (DKD) and to use bioinformatics methods to determine the potential mechanism in the pathological process of advanced DKD.

**Methods:**

A total of 178 type 1 diabetes mellitus (T1DM) patients with microalbuminuria complications who were hospitalized at least twice (with an interval > 24 months) in the Department of Endocrinology of The First Affiliated Hospital of USTC were included in this study. According to the urinary albumin creatinine ratio (UACR), we classified DKD stages as follows: microalbuminuria (UACR, 30-300 mg/g), and macroalbuminuria (UACR, >300 mg/g). We divided the patients into DKD progression (N=44) and stable group (N=134) on account of urinary albumin-to-creatinine ratio (UACR) by at least two randomized measurements. Stable group was defined as UACR between 30 and 300 mg/g, whereas progression group was defined as UACR >300 mg/g at the end of follow-up. Data were obtained from participants’ medical records, and the 25-hydroxyvitamin D level was categorized into three groups as follows: G1 (N=45), <10 ng/mL; G2 (N=80), 10-20 ng/ml; and G3 (N=53), ≥20 ng/mL. The Nephroseq database (http://v5.nephroseq.org) was used to identify VDR expression in diabetic nephropathy. The dataset GSE142025 from GEO (http://www.ncbi.nlm.nih.gov/geo) was downloaded. After stratification by the median-centered log2 VDR expression value, the 21 advanced DKD samples were divided into two groups (low VDR expression group and high VDR expression group). Gene set enrichment analysis (GSEA) (http://software.broadinstitute.org/gsea/index.jsp). Differentially expressed genes (DEGs) were screened by the limma package (adjusted p < 0.05, |logFC| > 1). The Gene Ontology (GO; http://www.geneontology.org/) database and pathway analysis within the Kyoto Encyclopedia of Genes and Genomes (KEGG; https://www.kegg.jp/) were performed using the R package ClusterProfile. The CIBERSORT (Cell type Identification By Estimating Relative Subsets Of known RNA Transcripts) algorithm was utilized for calculating the infiltrated immune cells in advanced kidney tissues.

**Results:**

1) A multivariate Cox regression analysis revealed that DR (diabetic retinopathy), eGFR (estimated glomerular filtration rate), and 25-hydroxyvitamin D were significant independent predictors of DKD progression (HR: 2.57, 95% CI: 1.44.4.24, p=0.007; HR: 2.13, 95% CI: 1.58.3.79, p = 0.011; HR: 0.732, 95% CI: 0.232–0.816, p = 0.023, respectively). 2) Kaplan–Meier survival curves of DKD progression by serum 25-hydroxyvitamin D stratification showed that the G2 and G3 groups were significantly different when compared with the G1 group (log-rank χ2 = 14.69, p <0.001; χ2 = 28.26, p <0.001, respectively). 3) There was a weak negative correlation between 25-hydroxyvitamin D level and UACR at baseline,and the overall mean rate of change in eGFR was 1.121 ± 0.19 ml/min/1.73 m2/year. Neither crude nor adjusted rate of decline in eGFR was significantly different among patients classified according to baseline serum 25-hydroxyvitamin D levels (all p<0.05). 4) The high expression of VDR group was most positively correlated with enriched gene sets like reactome innate immune system and reactome G alpha I signaling events when compared with the low expression of VDR group. 5) The CIBERSORT algorithm showed decreased M2 macrophage infiltration in advanced kidneys in comparison to low VDR expression and high VDR expression.

**Conclusion:**

This study concluded that low 25-hydroxyvitamin D levels can predict an increased risk of DKD albuminuria progression and eGFR decline. Decreased M2 macrophage infiltration may be a potential mechanism involved in this pathogenesis.

## Introduction

In most developed countries, diabetes is now the major cause of end-stage renal disease (ESRD) and is often responsible for >40% of new cases of ESRD (1). Several studies have reported that diabetic kidney disease (DKD) is related to the onset of cardiovascular diseases, which worsen the overall prognosis (2). Microalbuminuria is the first clinical symptom of patients with DKD, and uncontrolled microalbuminuria could evolve to macroalbuminuria and organ damage. Therefore, early detection and therapy are essential to delaying DKD progression, avoiding kidney function deterioration, and mitigating the burden of advanced disease.

Recently, the effects of reno-protective mediated by vitamin D receptor (VDR) activation have been demonstrated. Vitamin D and its analogues binding with VDR, which participates in a variety of pathophysiological processes, including inflammation, endothelial dysfunction, and upregulation of the renin–angiotensin–aldosterone system (RAAS) (3). The potential role of vitamin D in protection against DKD is of particular interest. Studies in both vitro and vivo have demonstrated a negative correlation between vitamin D levels and the risk of DKD (4). A previously published randomized controlled trial with a small sample size suggested an association between high-dose vitamin D supplementation and improvement in DKD stages in diabetes. Compared to that in the placebo group, the urinary albumin excretion rate was 18% lower in the vitamin D analog group (5).

However, there is no current consensus on the predictive value of low serum 25-hydroxyvitamin D levels for advanced DKD. In addition, the exact mechanism by which vitamin D is involved in the progression of DKD is unclear. In this study, we aimed to assess the correlation between serum 25-hydroxyvitamin D levels and DKD progression and used bioinformatics methods to determine the potential mechanism in the pathological process of advanced DKD.

## Materials and Methods

### Participants of Clinical

A total of 178 T1DM patients with microalbuminuria complications who were hospitalized at least twice (with an interval of > 24 months) in the Department of Endocrinology of the First Affiliated Hospital (Anhui Provincial Hospital) of the University of Science and Technology of China (baseline from July 2015 to September 2017) were considered for this study. The requirement for informed consent was waived, as this was a retrospective study of collected data available from participants’ medical records. According to the urinary albumin creatinine ratio (UACR), we classified DKD stages as follows: microalbuminuria (UACR, 30-300 mg/g), and macroalbuminuria (UACR, >300 mg/g). We divided the patients into DKD progression (N=44) and stable group (N=134) on the basis of urinary albumin-to-creatinine ratio (UACR) by at least two randomized measurements. Stable group was defined as UACR between 30 and 300 mg/g, whereas progression group was defined as UACR >300 mg/g at the end of follow-up (average follow-up time 43 months). The exclusion criteria were as follows: 1) patients with acute complications of diabetes; 2) history of vertebral fracture or osteoporosis and parathyroid conditions, including hypoparathyroidism and hyperparathyroidism; 3) patients with a history of taking vitamin D supplements or medications, such as anticonvulsants or systemic glucocorticoids; 4) presence of cancer, liver disease, or other co-existing conditions, including a history of coronary stenting, cerebral infarction, and severe CKD (defined as eGFR ≤60 mL/min/1.73 m^2^); and 5) other kidney diseases that may affect renal function and proteinuria, which increasing the chances of hospitalization. We excluded patients who last hospitalized were duo to acute kidney injury, urinary tract stones, urinary tract infection, primary glomerulonephritis, etc.

### Data of Clinical Participants

Data regarding the duration of diabetes, age, sex, body mass index (BMI, kg/m^2^), and use of RAAS inhibitor (RAASi) drugs (taken continuously for least 3 months) were obtained from the participants’ medical records. Additionally, blood pressure was recorded on admission. The 25-hydroxyvitamin D levels were assayed using a competitive binding radioimmunoassay technique (Bruxelles, Belgium). The seasons of 25-hydroxyvitamin D level measurement were classified as follows: winter (November to April) and summer (May to October). The UACR was tested twice, 1 or 2 days after administration and 1 or 2 days before discharge, and the mean value was calculated. The final diagnosis of diabetic retinopathy (DR) was made using fundus photographs, and the assessments were performed by two different ophthalmologists after training, respectively. All patients were tested for diabetes evaluation (HbA1c), routine blood tests (hemoglobin, Hs-CRP), biochemical data (ALT, AST, TBIL, BUN, Cr, and uric acid levels), lipid profile (TG, LDL-C, HDL-C, and TC levels), and 25-hydroxyvitamin D levels at baseline. The eGFR was calculated as follows: 194× Cr^-1.094^× Age^-0.287^ (×0.739 for female patients).

### Nephroseq Database Analysis

The Nephroseq database (6) (http://v5.nephroseq.org) was used to identify VDR expression in diabetic nephropathy. Correlation analysis and subgroup analysis between VDR and clinical features were also carried out to confirm the potential functions of VDR in DKD.

### GSE142025 Analysis

We downloaded the dataset GSE142025 from GEO (http://www.ncbi.nlm.nih.gov/geo). In the data set, only advanced DKD kidney samples were selected. After stratification by the median-centered log2 VDR expression value, the 21 samples were divided into two groups (low VDR expression group and high VDR expression group). Gene set enrichment analysis (GSEA) (7) (http://software.broadinstitute.org/gsea/index.jsp) is a kind of gene expression data based on molecular signatures database method. After 1000 permutation tests, the most significant pathways related to VDR expression in DKD were all selected as the critical p-value of significance level, the false discovery rate Q-value and the family wise error rate P-value. In this study, a total of 3323 genes (all adjusted p<0.05) were included. After consolidation and normalization of the RNA-seq data, 551 DEGs involved in advanced DKD patients were screened by the limma package (adjusted p < 0.05, |logFC| > 1). To understand the biological function and signaling pathways of the commonly shared DEGs involved in advanced DKD with different expression of VDR genes, the 551 identified DEGs were subjected to enrichment analysis within the Gene Ontology (8) (GO; http://www.geneontology.org/) database and pathway analysis within the Kyoto Encyclopedia of Genes and Genomes (9) (KEGG; https://www.kegg.jp/) using the R package ClusterProfile (Adjusted P values of <0.05 and Q-values <0.05). The CIBERSORT (10) (Cell type Identification By Estimating Relative Subsets Of known RNA Transcripts) algorithm, was applied to calculate the infiltrated immune cells in advanced kidney samples stratified by VDR expression level.

### Statistical Analysis

IBM SPSS Statistics ver. 22.0 (IBM Co., Armonk, NY, USA) was utilized, and two-tailed p <0.05 was set as statistical significance. Continuous measurements were described as the mean (SD) or the median (IQR) according to whether they were normally distributed or not. Categorical variables were described using frequencies (percentages). T tests, chi-square tests, or Mann–Whitney U tests, were used to compare the two patient groups. A Cox proportional hazards regression model to estimate the hazards ratio (HR) with a 95% class interval was used to analyse the risk factors for DKD progression or stability after adjusting for potential confounding variables. Kaplan–Meier survival curves of DKD progression by serum 25-hydroxyvitamin D level stratification were determined. Spearman’s correlation analysis was used to describe the relationship between 25-hydroxyvitamin D levels, UACR, and eGFR. Analysis of variance (ANOVA) and analysis of covariance (ANCOVA) were carried out to compare the rate of change in eGFR among the groups according to baseline serum 25-hydroxyvitamin D levels. Correlation analysis and subgroup analysis between VDR and clinical features *via* Nephroseq v5. data were carried out *via* an unpaired Student’s t test. The plots were generated by using GraphPad Prism (version 8.0; GraphPad Software, La Jolla, California).

## Results

### Demographic and Metabolism Characterization of Study Subjects

A total of 178 T1DM participants with microalbuminuria (51.69% male) were evaluated. Those patients had been diabetic for 0–41 years and mean age was 31.28 ± 14.79 years(age varied from 4 to 81 years). We divided the patients into DKD progression (N=44) and stable group (N=134) on the basis of urinary albumin-to-creatinine ratio (UACR) by at least two randomized measurements. Stable group was defined as UACR between 30 and 300 mg/g, whereas progression group was defined as UACR >300 mg/g at the end of follow-up (average follow-up time 43 months). Patients in the progression group had a significantly higher percentage of DR incidence and lower hemoglobin levels and TBIL, eGFR, and 25-hydroxyvitamin D levels than those in the stable group (both p <0.05). There were no significant differences in sex, age, BMI, follow-up time, blood pressure, duration of diabetes, HbA1c, hs-CRP, ALT, AST, BUN, Cr levels, or lipid profile between the two groups (all p >0.05) (**Table 1**).

**Table 1:** Demographic and metabolism characterization of study subjects.

Group	Progression group (N = 44)	Stable group (N = 134)	T/Z/χ2 value	P value
**General data**				
Gender (Male/Female)	24/20	68/66	0.191	0.662
Age (year)	31.98 ± 15.09	31.04 ± 14.74	-0.362	0.718
BMI (kg/m^2^)	21.25 ± 3.55	20.56 ± 3.82	1.060	0.291
Average follow-up time (month)	42 (36-44)	43 (37-50.75)	-1.366	0.172
Diabetic retinopathy (number,%)	18 (40.91%)	31 (23.13%)	5.246	0.022
Systolic blood pressure (mmHg)	120.34 ± 15.04	118.81 ± 15.38	-0.576	0.566
Diastolic blood pressure (mmHg)	76.73 ± 9.19	74.71 ± 11.03	-1.096	0.275
RAASi usage (number,%)	21 (47.73%)	57 (42.54%)	0.362	0.547
**Glucose metabolism**				
Duration of diabetes (year)	6.5 (3-12)	5 (2-10)	-1.447	0.148
HbA1c (%)	10.40 ± 4.77	9.01 ± 2.33	-1.853	0.070
UACR (mg/gCr)	74 (41-198)	67 (39-221)	-1.564	0.112
**Routine blood test**				
Hemoglobin (g/L)	133.35 ± 14.16	138.24 ± 12.41	-2.189	0.030
hs-CRP (mg/L)	5.02 ± 1.09	4.69 ± 1.18	-1.639	0.103
**Biochemical data**				
ALT (IU/L)	17 (14–23)	16 (11–23)	-0.228	0.653
AST (IU/L)	15(9-24)	16 (8-24)	0.105	0.893
TBIL (µmol/L)	9.24 ± 2.03	12.08 ± 2.65	6.928	<0.001
BUN (mmol/L)	5.03 ± 1.59	5.43 ± 2.06	1.156	0.249
Cr (µmol/L)	62.58 ± 17.23	58.37 ± 15.43	-1.525	0.129
eGFR (mL/min/1.73 m`2)	92.84 (77.66-152.79)	114.13 (93.56-136.86)	-2.320	0.020
Uric acid (umol/L)	247.78 ± 92.43	246.79 ± 79.41	-0.069	0.945
Calcium (mmol/L)	2.37 ± 0.24	2.39 ± 0.25	0.465	0.643
25-hydroxyvitamin D (ng/ml)	9.41(6.28-13.71)	17.74 (11.99-22.91)	-5.69	<0.001
Season of measurement (winter/summer)	23/21	64/70	0.270	0.603
**Lipid profile**				
TG (mmol/L)	1.03 (0.74-1.67)	0.92 (0.63-1.33)	-1.721	0.850
TC (mmol/L)	4.33 (3.59-5.13)	4.31 (3.65-5.16)	-0.094	0.925
LDL-c (mmol/L)	2.39 (1.66-2.81)	2.19 (1.65-2.93)	-0.201	0.841
HDL-c (mmol/L)	1.34 (1.08-1.79)	1.31 (1.09-1.62)	-0.651	0.515

Data are n (%), mean ± SD, or median (interquartile range).

### Independent Risk Factors Associated With the Progression of DKD

**Figure 1:**
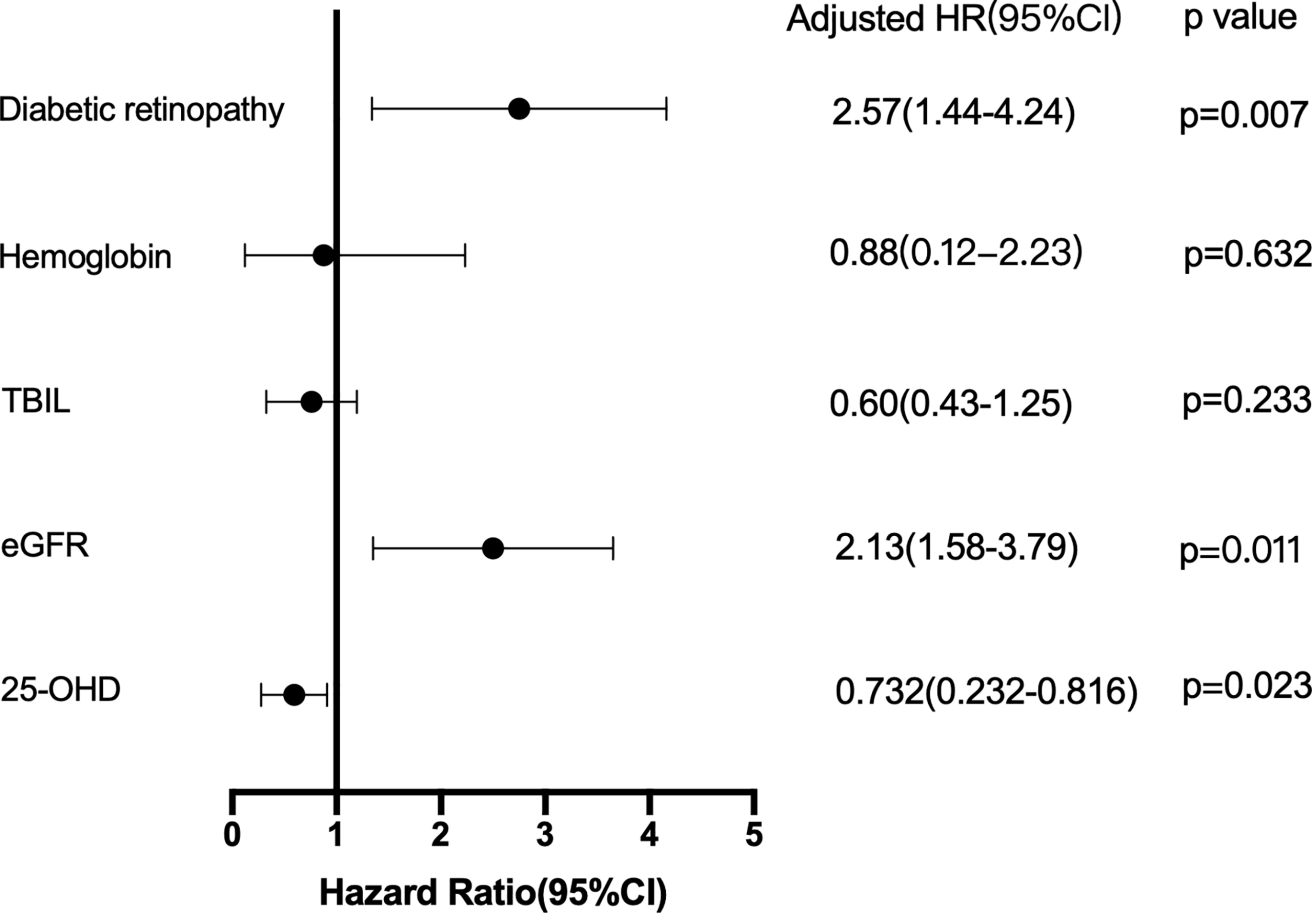
Cox proportional hazards regression model: the risk factors for DKD progression.

Cox proportional risk regression model was used to analyze the risk factors of DKD progression. After adjusting for all factors that appeared significantly in the univariate analysis, DR, eGFR, and 25-hydroxyvitamin D levels were significant independent predictors of DKD progression (HR: 2.57, 95% CI: 1.44.4.24, p=0.007; HR: 2.13, 95% CI: 1.58.3.79, p = 0.011; HR: 0.732, 95% CI: 0.232–0.816, p = 0.023, respectively), as presented in **Figure 1**.

### The End Point of DKD Progression by Serum 25-Hydroxyvitamin D Stratification

**Figure 2:**
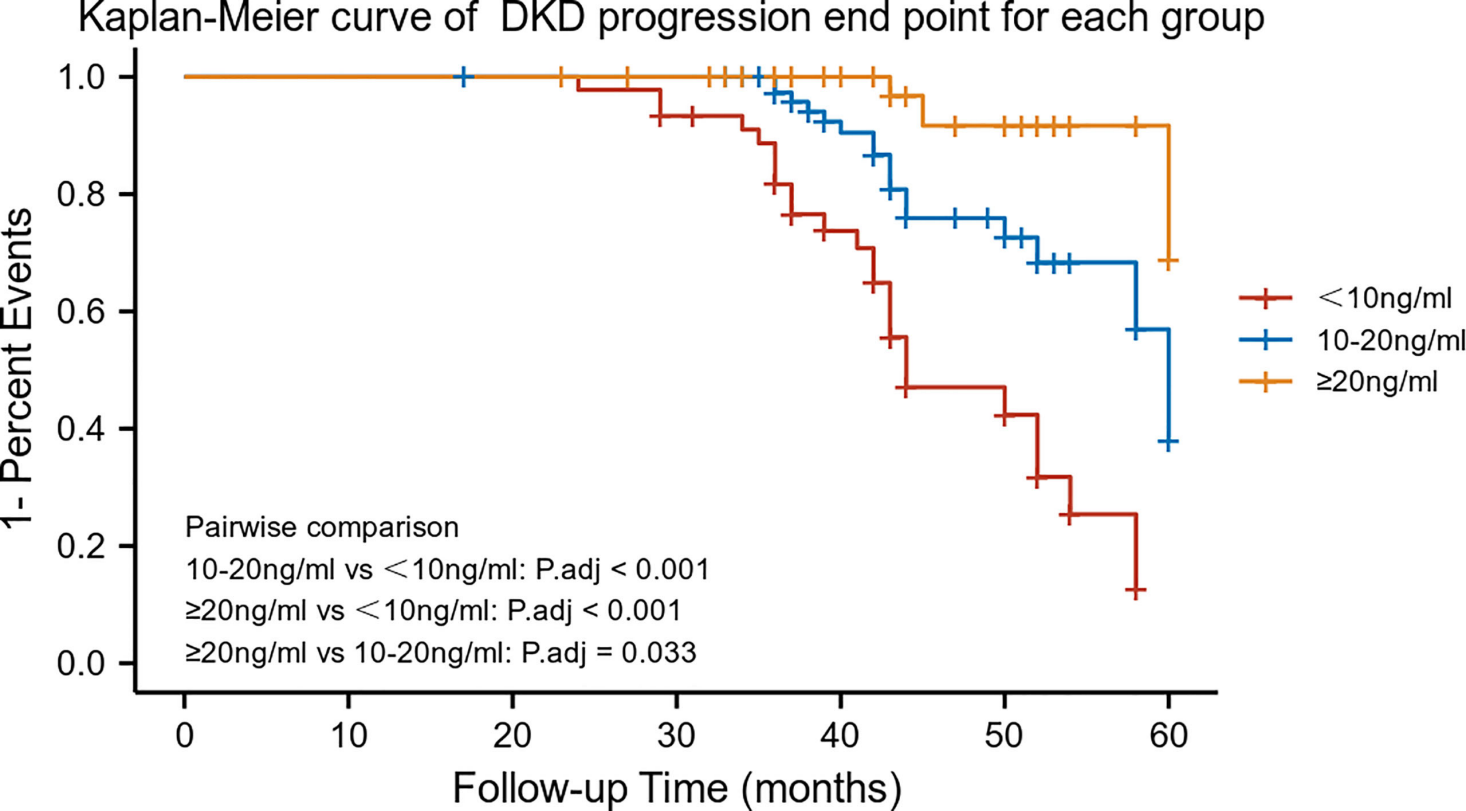
Kaplan–Meier survival curves by 25-hydroxyvitamin D stratification (n = 178). Patients were categorized into three groups: G1 (N = 45); G2 (N = 80); and G3 (N = 53).

As mentioned above, serum 25-hydroxyvitamin D levels were correlated with DN progression outcome. Further analysis with 25-hydroxyvitamin D level stratification was performed to estimate the ratio of different serum 25-hydroxyvitamin D levels. The 25-hydroxyvitamin D levels were categorized into three groups as follows: G1 (N=45), <10 ng/mL; G2 (N=80), 10-20 ng/ml; and G3 (N=53), ≥20 ng/mL. The G2 and G3 groups were significantly different when compared with the G1 group (log-rank χ2 = 14.69, p <0.001; χ2 = 28.26, p <0.001, respectively) (**Figure 2**).

### Relationship Between 25-Hydroxyvitamin D, UACR and eGFR

**Figure 3:**
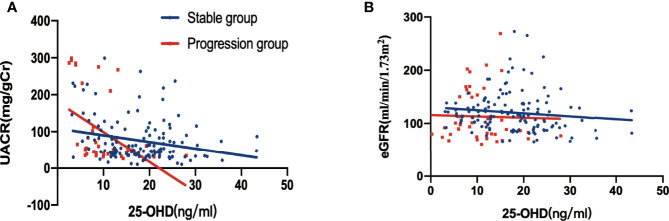
Relationship between 25-hydroxyvitamin D, UACR and eGFR at baseline, stratified by DKD albuminuria progression. **(A)** Correlation between 25-hydroxyvitamin D and UACR (p=0.0038 in the stable group, p = 0.0011 in the progression group); **(B)** correlation between 25-hydroxyvitamin D and eGFR in **Figure 3B** (all p > 0.005).

There was a negative correlation between 25-hydroxyvitamin D levels and UACR at baseline (stable group, r=-0.2487, p=0.0038; progression group, r=-0.4771, p=0.0011) (**Figure 3A**). However, there was no significant relationship between baseline 25-hydroxyvitamin D levels and eGFR, regardless of the group (all p>0.05) (**Figure 3B**).

### Comparison of Rate of Change in eGFR Among Groups According to Baseline Serum 25-Hydroxyvitamin D Levels

**Figure 4:**
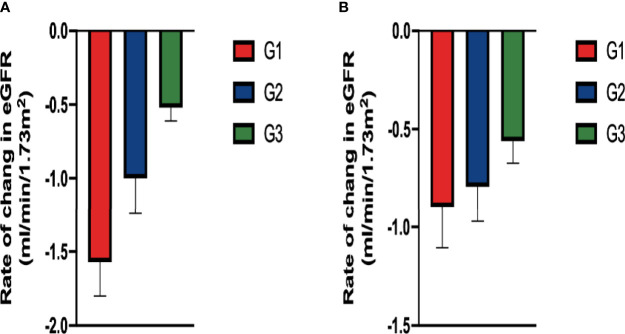
Comparison of rate of change in eGFR among groups classified according to baseline serum 25-hydroxyvitamin D levels: In **(A)** the crude model using ANOVA and in **(B)** the adjusted model (After adjusting for diabetic retinopathy, hemoglobin and TBIL in baseline, ANCOVA). There were significant difference in the rate in the both crude and adjusted model.

The overall mean rate of change in eGFR was 1.121 ± 0.19 ml/min/1.73 m^2^/year. Both crude and adjusted rates of decline in eGFR were significantly different among patients classified according to baseline serum 25-hydroxyvitamin D levels (Crude, G1: -1.57 ± 0.23, G2: -1.00 ± 0.24, G3: -0.521 ± 0.09, p<0.001, p for trend=0.015;Adjusted, G1:-0.896 ± 0.21, G2: -0.793 ± 0.178, G3:-0.561 ± 0.112, p<0.001, p for trend=0.042) (**Figure 4**).

### The Expression of VDR in DKD Renal Tissue Samples

**Figure 5:**
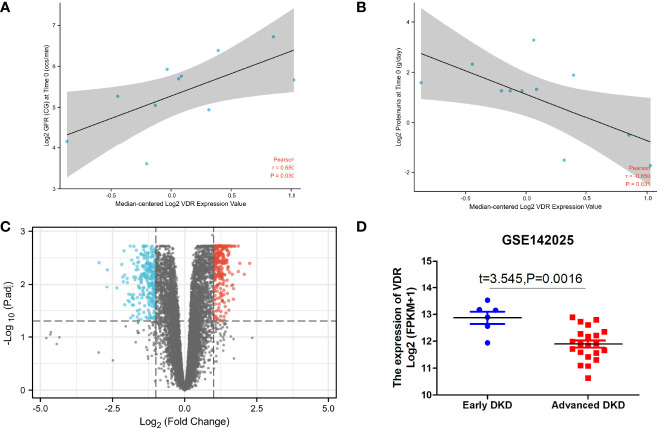
VDR in DKD renal tissue samples. The correlation between VDR expression and eGFR **(A)** and proteinuria **(B)** of DKD patients with the use of Nephroseq v5. **(C)** Differential gene expression analysis for the low VDR expression group vs. the high VDR expression group. **(D)** The expression of VDR in early DKD samples vs. advanced DKD samples.

The expression of VDR showed a difference between DKD and non-DKD mice in Nephroseq v5 datasets (**Figure 5**). In addition, the correlation between VDR expression and eGFR and proteinuria of DKD patients was determined (**Figures 5A, B**). The expression of VDR in DKD renal tissue samples was positively correlated with eGFR but negatively correlated with proteinuria. Thus, the decreased expression of VDR may result in the occurrence and development of DKD. To identify DEGs linked with VDR gene expression in DKD, we downloaded relevant expression profiles from GSE142025. After consolidation and normalization of the RNA-seq data, 551 DEGs involved in advanced DKD patients were screened by the limma package (adjusted p < 0.05, |logFC| > 1), as shown in the volcano plot (**Figure 5C**). The expression of VDR in early DKD renal samples was significantly higher than that in the advanced DKD group (**Figure 5D**).

### GSEA Stratified by Median-Centered log2 VDR Expression Value

**Figure 6:**
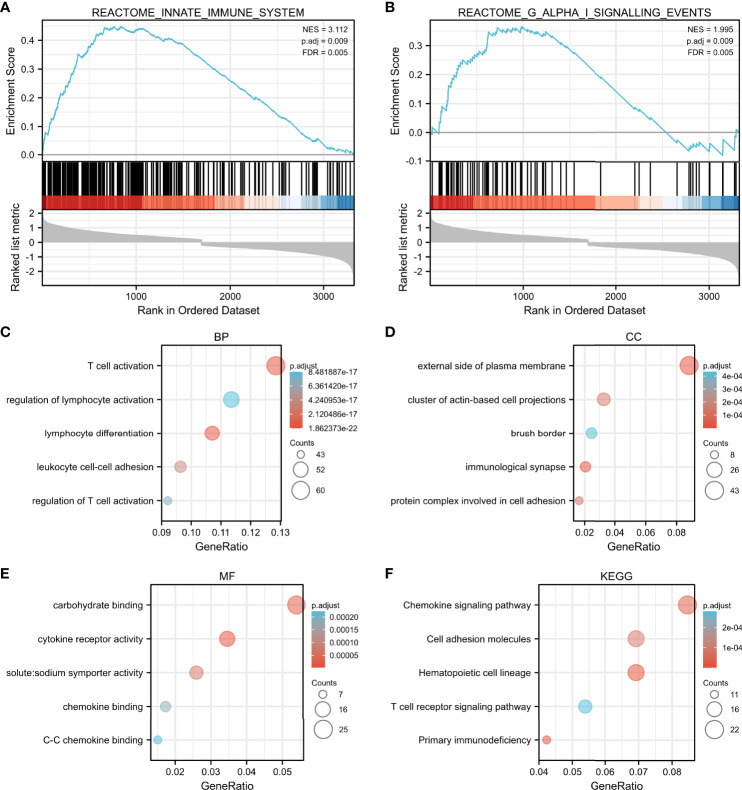
GSEA and functional enrichment by median-centered log2 VDR expression value. The high expression of VDR group was most correlated with enriched gene sets such as reactome innate immune system **(A)** and reactome G alpha I signaling events **(B)**. Enriched gene ontology (GO) functions of the 551 DEGs according to three complementary biological roles: biological process **(C)**, cellular component **(D)** and molecular function **(E)**. KEGG enrichment result of DEGs were shown in **(F)**.

The high expression of VDR group was most positively correlated with enriched gene sets like reactome innate immune system (**Figure 6A**) and reactome G alpha I signaling events (**Figure 6B**) when compared with the low expression of VDR group.

### Functional Annotation and Enrichment

To investigate the biological function of the 551 identified DEGs involved in advanced DKD with different VDR expression, a gene ontology (GO) enrichment analysis was undertaken. The response to T cell activation was the most significantly enriched GO term of BP (**Figure 6C**). In terms of CC ontology, the external side of the plasma membrane was significantly enriched (**Figure 6D**). The most significantly enriched MF was carbohydrate binding (**Figure 6E**). In our KEGG enrichment analysis, “chemokine signaling pathway”, “cell adhesion molecules”, “hematopoietic cell lineage”, “T cell receptor signaling pathway”, and “primary immunodeficiency” were significantly enriched KEGG pathways (**Figure 6F**).

### The Relationship Between VDR Expression and Immune Cell Infiltration

**Figure 7:**
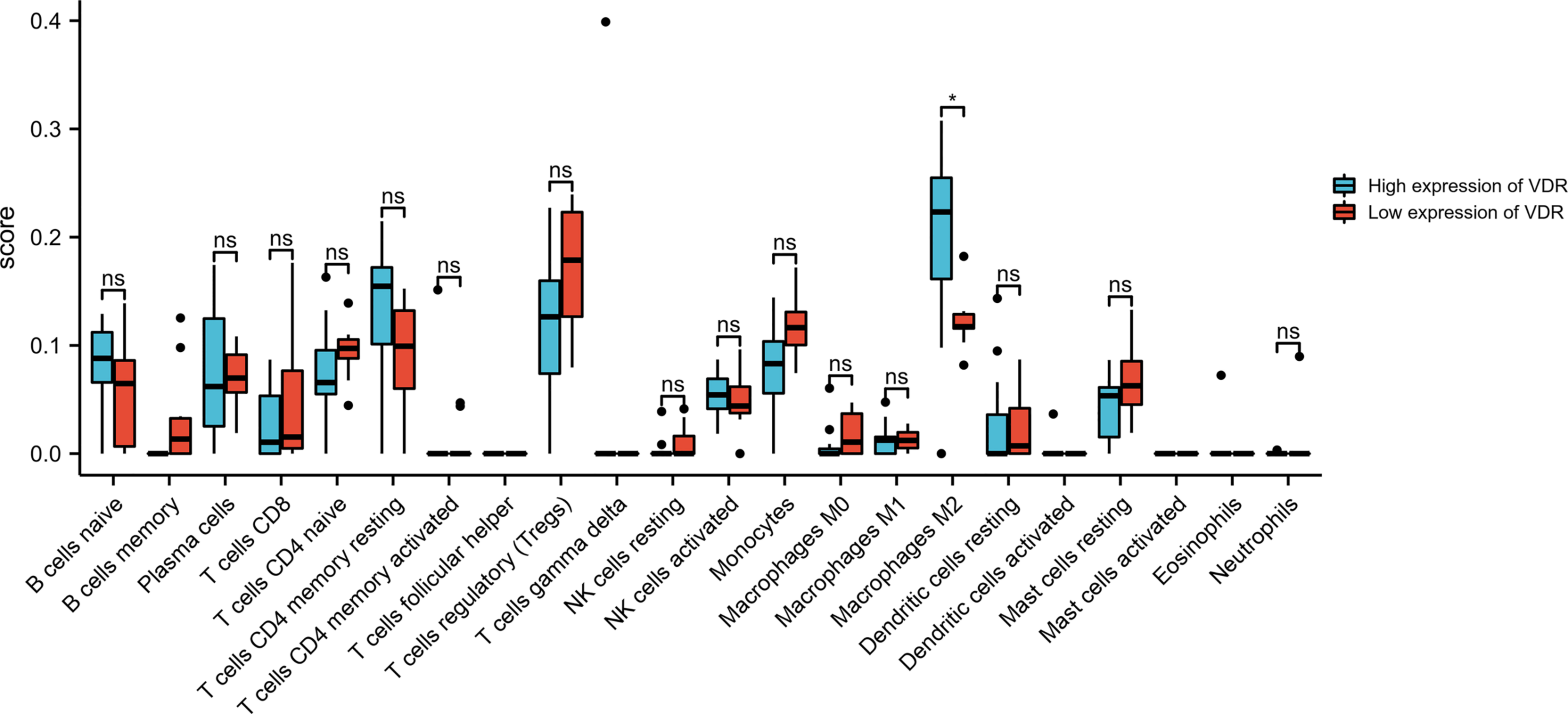
Bar plot showing the difference between 22 infiltrated immune cells in the advanced DKD samples stratified by VDR expression level with the CIBERSORT algorithm. ns, no significance.

The results above suggest that the regulation of immune-related pathways by VDR may play a vital role in the DKD progression. Thus, understanding the relationship between VDR expression and immune cell infiltration may provide a more comprehensive view of advanced DKD therapy efficacy. The CIBERSORT algorithm showed decreased M2 macrophage infiltration in advanced kidneys in comparison to low VDR expression and high VDR expression (**Figure 7**).

## Discussion

Previous studies have shown that 25-hydroxyvitamin D levels play a protective role against the development of diabetic microvascular complications. To the best of our knowledge, this is the first study to investigate the predictive value of serum concentration of 25-hydroxyvitamin D in the risk of DKD progression. In the Cox proportional hazards regression analysis, DR, eGFR, and 25-hydroxyvitamin D levels were independently related to DKD progression. Subsequent Kaplan–Meier survival analysis stratified by 25-hydroxyvitamin D levels indicated that the G2 and G3 groups were significantly different in the incidence of DKD progression compared to the G1 group. Further analysis indicated that after adjusting for diabetic retinopathy, hemoglobin, and TBIL at baseline, the rate of decline in eGFR was significantly different among patients classified according to baseline serum 25-hydroxyvitamin D levels. Bioinformatics analysis shows that the regulation of immune-related pathways by VDR may play a vital role in the DKD progression. The CIBERSORT algorithm showed decreased M2 macrophage infiltration in advanced kidneys in comparison to low VDR expression and high VDR expression. These findings may provide assistance for assessing prognosis and determining treatment choice in DKD patients.

In recent years, diabetes and its complications pose a heavy burden to people and have a significant impact on health and quality of life (11). DKD is a slow progressive condition and a major cause of kidney failure worldwide. Epidemiological studies have shown that 25% to 40% of individuals with T1DM and 5% to 40% of people with type 2 diabetes (T2DM) ultimately develop DKD (12). It is important to identify and monitor diabetic patients with microalbuminuria, as treatment can prevent or postpone overt nephropathy. However, most individuals with early-stage DKD (microalbuminuria) have low rates of awareness, attention, and treatment (13). Once microalbuminuria progresses, it is often too late to alleviate adverse patient outcomes. Consequently, it is essential to identify predictive factors for progression during the early course of DKD.

Vitamin D deficiency (VDD) has been shown to be involved in a variety of pathophysiological processes, such as endothelial dysfunction, inflammation and RAAS upregulation, all of which are associated with the occurrence of diabetic complications. Among them, the potential of vitamin D in the protection of DKD has attracted special attention (14–16). A meta-analysis conducted by Feng et al. in 2015 with a total sample size of 3494 participants (1790 controls and 1704 T1DM) and shown the high vitamin D deficiency rates in patients with T1DM (17). Additionally, VDD has been shown to be involved in several diabetic complications including diabetic peripheral neuropathy, retinopathy, and DKD (18–21). DKD progression exacerbates the insufficiency of vitamin D (22). In line with this, De Boer et al. investigated the relationship between serum vitamin D and microalbuminuria in T1DM patients, which found that low concentrations of 25-hydroxyvitamin D (below 20 ng/mL) were associated with an increased risk of microalbuminuria in 65% of patients (23). Fernandez-Juarez et al. recruits 133 T2DM patients, all of whom had preexisting albuminuric CKD. Using a composite endpoint (serum creatinine >50% increase, ESRD, and mortality), they found that low vitamin D levels were independently associated with DKD progression (24).

However, the results of observational cohort studies in individuals with diabetes have been inconsistent in defining the relationship between low 25-hydroxyvitamin D levels and microalbuminuria. Engelen et al. found that low 25-hydroxyvitamin D levels are independently associated with macroalbuminuria but not with microalbuminuria in T1DM (25). Fawzy et al. suggested a possible clinical application of urinary vitamin D-binding protein as a good early marker for the detection of early kidney disease in T2DM, but the predictive value of serum 25-hydroxyvitamin D levels was not mentioned (26). Low 25-hydroxyvitamin D levels are associated with decreased eGFR in patients with T2DM, especially in older patients, with no evidence of interaction with UACR levels (27). Similarly, the results regarding the value of vitamin D or its analog treatment in DKD patients remain inconsistent; Chokhandre showed that vitamin D and its analogs considerably improve renal function by reducing UACR and eGFR in patients with CKD (28). The addition of calcitriol safely resulted in a significant reduction in albuminuria by randomized controlled trial (29). However, a systematic meta-analysis of nine trials suggested that despite a higher risk of DKD in vitamin D-deficient patients, vitamin D supplementation did not lead to significant changes in UACR (30). Heng showed that the protective effects of 25-hydroxyvitamin D on the kidney may start in the very early stage of diabetes, but there is no association between 25-hydroxyvitamin D levels and reduced eGFR (27). Our results show that lower 25-hydroxyvitamin D levels predict an increased risk of DKD progression, indicating that serum 25-hydroxyvitamin D concentration is negatively correlated with the development of DKD and may be a useful predictor of serious progression of DKD in the early stage.

Both DR and DKD are microvascular complications of diabetes mellitus, and they are often complicated. Patients with DR had a 31% increased risk of DKD (31). Our study also demonstrated that DR and lower eGFR are relevant to higher progression rates in microalbuminuria subjects who later progress to macroalbuminuria. Studies have reported that vitamin D deficiency can induce endothelial dysfunction *via* inflammation and upregulation of the RAAS. A study by Forman et al. indicated a negative relationship between 25-hydroxyvitamin D and plasma angiotensin II levels in normotensive individuals (32). Another large cohort study suggested that serum levels of 25-hydroxyvitamin D were independently and inversely correlated with the concentrations of renin and angiotensin II (33). Further mechanistic studies have shown that vitamin D decreases the expression of renin by inhibiting the promoter activity of the renin gene and suppresses the high glucose-induced expression of angiotensinogen by blocking the nuclear factor-kappa B (NF-κB) signaling pathway (34). The levels of renin and angiotensin were shown to be significantly decreased in vitamin D-supplemented diabetic rats (35). Another study has shown that vitamin D can inhibit the activation of the RAAS and hyperglycemia-induced production of TGF-β (transforming growth factor-β), thus decreasing the number of TGF-β-stimulated renal interstitial fibroblasts and inhibiting renal tubular epithelial-mesenchymal transdifferentiation (36). The increase in angiotensin II levels under high-glucose conditions activates NF-κB, which in turn supports the accumulation of angiotensin II, creating a positive feedback loop that exacerbates kidney injury (37). However, in our study, even though the baseline data for the utilization rate of RAASi were uniform across the two groups, a more pronounced albuminuria progression and an expedited decline of renal function in the lower concentration of serum 25-hydroxyvitamin D were observed, suggesting that vitamin D may be involved in other potential mechanisms, in addition to the RAAS pathway.

Increasing evidence has demonstrated that the activation of VDR signaling pathway exerts a variety of reno-protective effects in DKD patients, such as anti-inflammatory, anti-proteinuria, anti-fibrosis, and prevents podocyte damage (38). However, the effects and mechanisms still need to be further explained. Macrophages participate in different pathological stages of kidney injury, including exudation, recovery, and fibrosis. Among them, M1 macrophages dominate the proinflammatory effect, and M2 macrophages (alternatively activated macrophages) dominate the anti-inflammatory response and tissue repair (39). M2 macrophages have regenerative properties and show promise as a cell therapy in chronic kidney disease. However, M2 plasticity is one of the major hurdles to overcome. Our bioinformatics analysis shows decreased M2 infiltration in advanced kidneys in comparison to low VDR expression and high VDR expression, indicating that genetically modified macrophages stabilized by VDR were able to preserve their M2 phenotype and may provide a determining treatment choice in DKD patients.

Further prospective investigations of clinical and basic research are necessary to explore the causal associations and molecular mechanisms of vitamin D and advanced DKD. This study has several limitations as follows. First, considering ethnic and regional differences, this is a single center study in China and the results may not be directly applicable. Second, Unmeasured confounders may not be adequately addressed. Finally, the mechanisms by which VDR plays are not completely clear. More evidence is required to determine the biological foundation.

## Conclusions

This study concludes that low 25-hydroxyvitamin D levels predict an increased risk of DKD albuminuria progression and eGFR decline. Decreased M2 macrophage infiltration may be a potential mechanism involved in this pathogenesis.

## Data Availability Statement

The datasets presented in this study can be found in online repositories. The names of the repository/repositories and accession number(s) can be found in the article/supplementary material.

## Ethics Statement

Ethical review and approval was not required for this study with human participants, in accordance with the local legislation and institutional requirements.

## Author Contributions

BH performed the data acquisition and drafted the work. The main statistical work was completed by WW. SY and BH interpreted the patient data. SY substantively revised it. All authors read and approved the final manuscript.

## Funding

This study was supported by the National Natural Science Foundation of China (81800713) and the local scientific and technological development project guided by the central government of China (no. 2017070802D147).

## Conflict of Interest

The authors declare that the research was conducted in the absence of any commercial or financial relationships that could be construed as a potential conflict of interest.

## Publisher’s Note

All claims expressed in this article are solely those of the authors and do not necessarily represent those of their affiliated organizations, or those of the publisher, the editors and the reviewers. Any product that may be evaluated in this article, or claim that may be made by its manufacturer, is not guaranteed or endorsed by the publisher.
